# CRISPR/Cas9-mediated targeted mutation reveals a role for *AN4* rather than *DPL* in regulating venation formation in the corolla tube of *Petunia hybrida*

**DOI:** 10.1038/s41438-021-00555-6

**Published:** 2021-06-01

**Authors:** Bin Zhang, Xiaojing Xu, Renwei Huang, Sha Yang, Mingyang Li, Yulong Guo

**Affiliations:** 1 Chongqing Engineering Research Center for Floriculture, Key Laboratory of Horticulture Science for Southern Mountainous Regions, Ministry of Education, College of Horticulture and Landscape Architecture, Southwest University, 400716 Chongqing, China; 2 College of Agriculture, Guizhou University, 550025 Guiyang, Guizhou China; 3 Sichuan Provincial Key Laboratory for Development and Utilization of Characteristic Horticultural Biological Resources, College of Chemistry and Life Sciences, Chengdu Normal University, 611130 Chengdu, China

**Keywords:** Plant molecular biology, Molecular engineering in plants

## Abstract

Venation is a common anthocyanin pattern displayed in flowers that confers important ornamental traits to plants. An anthocyanin-related R2R3-MYB transcription factor, DPL, has been proposed to regulate corolla tube venation in petunia plants. Here, however, we provide evidence redefining the role of *DPL* in petunia. A CRISPR/Cas9-mediated mutation of *DPL* resulted in the absence of the vein-associated anthocyanin pattern above the abaxial surface of the flower bud, but not corolla tube venation, thus indicating that *DPL* did not regulate the formation of corolla tube venation. Alternately, quantitative real-time PCR analysis demonstrated that the spatiotemporal expression pattern of another R2R3-MYB gene, *AN4*, coincided with the formation of corolla tube venation in petunia. Furthermore, overexpression of *AN4* promoted anthocyanin accumulation by increasing the expression of anthocyanin biosynthesis genes. CRISPR/Cas9-mediated mutation of *AN4* led to an absence of corolla tube venation, suggesting that this gene in fact determines this key plant trait. Taken together, the results presented here redefine the prime regulator of corolla tube venation, paving the way for further studies on the molecular mechanisms underlying the various venation patterns in petunia.

## Introduction

Anthocyanin pigments contribute to the diverse colors and pigmentation patterns of flowers, which are important ornamental characteristics of horticultural plants. Anthocyanins are derived from the flavonoid biosynthetic pathway, involving a series of enzymes encoded by several structural genes^1^. In dicot plants, these structural genes can be subdivided into early biosynthetic genes (EBGs), including *chalcone synthase* (*CHS*), *chalcone isomerase* (*CHI*), *flavanone 3-hydroxylase* (*F3H*), and *flavonoid 3*′*-hydroxylase* (*F3’H*), and late biosynthetic genes (LBGs), such as *flavonoid 3*′*5*′*-hydroxylase* (*F3*′*5*′*H*), *dihydroflavonol 4-reductase* (*DFR*), *anthocyanidin synthase* (*ANS*), and *glutathione-S-transferase* (*GST*)^2–5^. Expression of these EBGs is regulated by R2R3-MYB transcription factors^6,7^, while the expression of the LBGs is regulated by the ternary complex MYB–bHLH–WD40 consisting of R2R3-MYB, a basic helix-loop-helix (bHLH), and WD40 transcription factors^8,9^. Among these transcription factors, R2R3-MYB genes play major roles in providing the required specificity for LBG expression and consequently determining the spatiotemporal accumulation of anthocyanin in plants^10,11^.

Venation patterning arises from pigmented stripes overlying veins and is a common anthocyanin pattern seen in flowers^12^. In *Antirrhinum*, the bHLH gene *Delila* is expressed in its petal epidermis, and *Venosa*, an R2R3-MYB gene, is specifically expressed in cells above the vascular tissues, with venation arising in the overlapping expression domains of the R2R3-MYB and bHLH genes^13^. Since the bHLH and WD40 genes are usually expressed constitutively, the formation of venation patterning depends mainly on *R2R3-MYB*, which provides vein specificity. For example, in *venosa* mutants, no venation is displayed in their petals^14^, and silencing of *PeMYB12*, an R2R3-MYB gene, induces the loss of venation patterning in sepals/petals of *Phalaenopsis* spp.^15^. In addition to venation, R2R3-MYB is also the key regulator of two other anthocyanin patterns: blotch^16^ and spot^17,18^.

*Petunia hybrida*, distinguished by its diverse flower colors and pigmentation patterns, is one of the most popular bedding plants^19^. In such petunia plants, diverse types of venation patterning have been developed in the corolla of some cultivars to improve their ornamental value. Several genetic loci reportedly control the various patterns of venation in the corolla of petunias. For example, individuals harboring the *Venation-1* (*Ve1*) locus display reticulate venation of the corolla tube, whereas *ve1* individuals exhibit a few longitudinal pigmented stripes over the veins; *Venation-2* (*Ve2*) and *Venation-3* (*Ve3*) loci determine the extension of venation from the corolla throat to the outer edge of the limb; and the *Fine venation* (*Fn*) locus controls the thickness of venation in the corolla limb^20^. A previous study proposed that an anthocyanin-related R2R3-MYB gene, *DEEP PURPLE* (*DPL*), may reside at the *Ve1* locus^21^. The role of *DPL* in the regulation of venation patterning in corolla tubes has since been cited widely in many recent publications^12,15,22–24^. In addition to *DPL*, another anthocyanin-related R2R3-MYB gene, *ANTHOCYANIN4* (*AN4*), is capable of associating with these *Ve* and *Fn* loci according to genetic analyses. For example, a stronger venation is associated with the presence of the dominant allele of *AN4* and the recessive *ve2* allele^20,25^. We know that *AN4* can regulate the anthocyanin accumulation in anthers^26^, but whether this gene also determines venation patterning remains unknown.

In this work, we reinvestigated the respective roles of *DPL* and *AN4* in flower development, mainly by using CRISPR/Cas9 targeted mutation technology. We found that venation patterning in petunia corolla tubes is actually regulated by *AN4*, not the proposed *DPL*. Our results also revealed that *DPL*’s true role in petunia flower petals is to regulate their vein-associated anthocyanin patterns above the abaxial surface.

## Results

### CRISPR/Cas9-induced mutation of *DPL* in MD

**Fig. 1:**
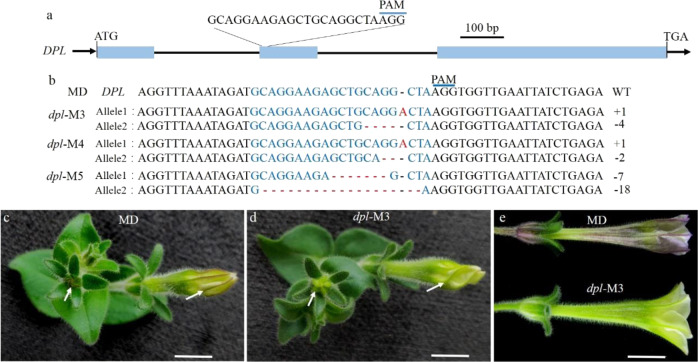
CRISPR/Cas9-mediated mutation in *DPL*. **a** Diagram of the target site in the genomic region of *DPL*. The blue line indicates the PAM (NGG) motif. The blue boxes and black lines indicate exons and introns, respectively. **b** Targeted mutagenesis in *DPL* for the *dpl*-M3, -M4, and -M5 lines. The target sequence is shown in blue letters, for which red dashes indicate deletions, while red letters indicate insertions. **c**–**e** Flower buds of MD and a representative line *dpl-*M3; flower buds in **c** and **d** are from plants cultured at 25 °C, and those in **e** are from plants treated at 12 ± 2 °C (cold stress conditions). Scale bars = 1 cm

To investigate the specific role of *DPL* in petunia, we mutated *DPL* in the *P. hybrida* inbred line “Mitchell Diploid’ (MD) by using the CRISPR/Cas9 system. One target site in the second exon was selected as the sgRNA complementary site (Fig. 1a), and three *DPL* biallelic mutants (*dpl*-M3, -M4, and -M5) in MD were obtained. In the *dpl*-M5 line, one allele of *DPL* exhibited a deletion of 18 bp, which introduced a new stop codon in *DPL*, leading to premature terminations. The deletion in the second *DPL* allele of *dpl*-M5 and the indel mutations in the *DPL* alleles of *dpl*-M3 and dpl-M4 mutants interrupted the reading frame, leading to frameshift mutations (Fig. 1b). Considering that the target sequence of *DPL* is highly similar to that in *AN4I* and *AN4II*, a potential off-target site was detected in *AN4I* and *AN4II* of the *dpl*-M3, -M4, and -M5 lines; however, no mutations could be found in the off-target site of these lines (Fig. S3a). Therefore, *dpl*-M3, -M4, and -M5 were *dpl*-knockout mutants and were used for further investigations.

No significant changes in corolla tube venation patterning were detected between *dpl* mutants and MD (Fig. S1a–d). However, vein-associated anthocyanin deposition above the abaxial surface of the flower bud was lost in *dpl* mutants (Fig. 1c, d and Fig. S1e–g). In the T1 lines of *dpl*-M3, -M4, and -M5, this mutated phenotype was observed. Vein-associated anthocyanin patterning initially emerged at the flower bud abaxial surface, but gradually faded as the flower opened (Fig. S1h). Furthermore, this anthocyanin patterning became more obvious in flower buds of MD, when these plants with flower buds were treated at 12 ± 2 °C for 2 weeks, but it was always absent in those of *dpl*-knockout mutants (Fig. 1e). These results indicated that *DPL* determines vein-associated anthocyanin occurrence above the abaxial epidermis of petunia flower buds rather than the venation patterning of its corolla tube.

### Positive correlation between the expression patterns of *AN4* and the formation of corolla tube venation

**Fig. 2:**
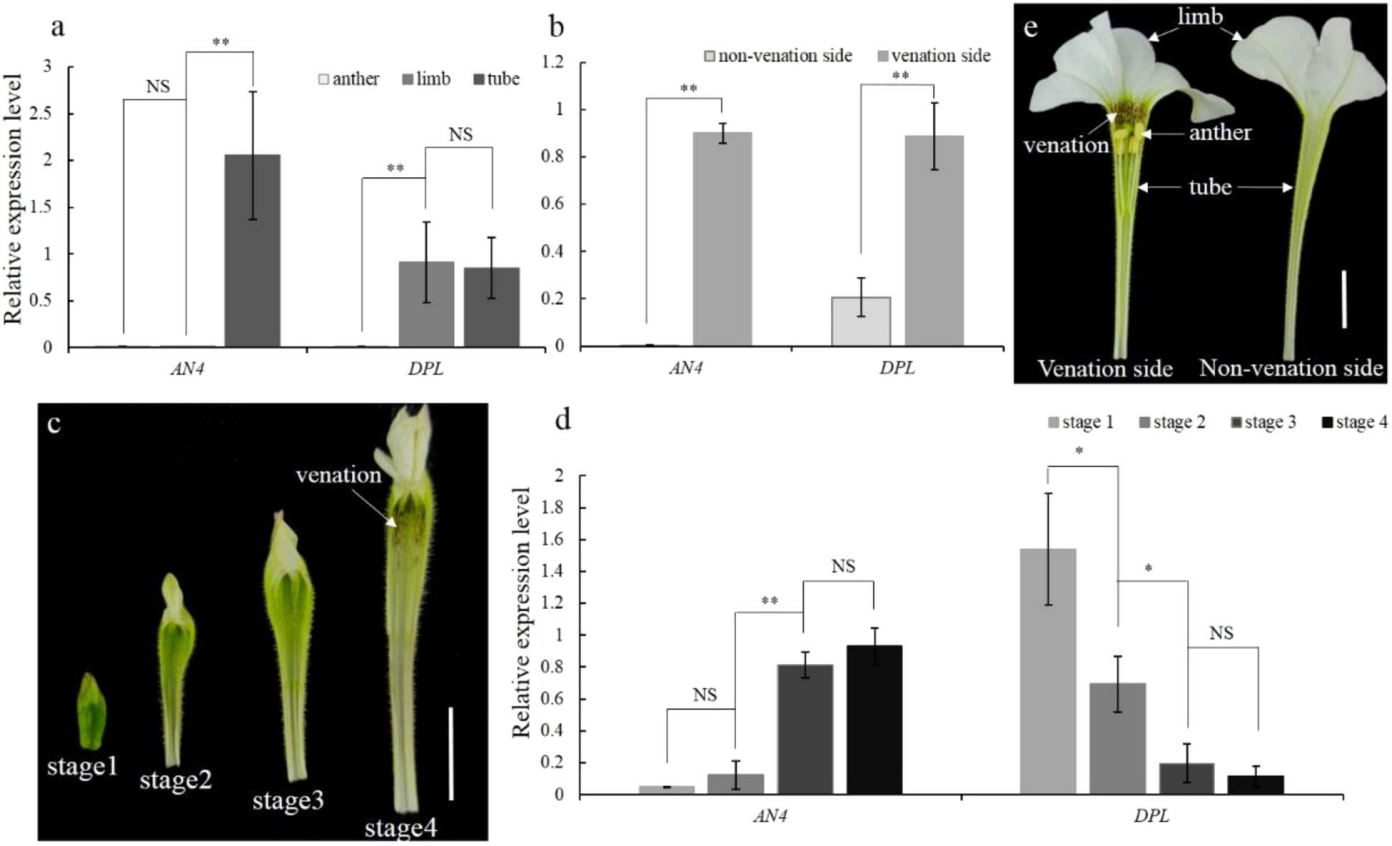
AN4 and DPL expression patterns. **a**, **b** Spatial expression pattern of *AN4* and *DPL* in the opening flower of MD. **c** Annotation of flower developmental stages in MD; scale bar = 1 cm. **d** Temporal expression patterns of *AN4* and *DPL* during flower development in MD. *SAND* was used as the reference gene. Shown are the means ± SDs, *n* = 3. Asterisks indicate a significant difference: *P < 0.05; **P < 0.01; nonsignificant (NS). **e** Annotation of different parts of the flower in MD; scale bar = 1 cm

Because venation patterning was not affected in *dpl* mutants, we next investigated whether other MYB genes were perhaps involved in regulating petunia venation patterning. qPCR analysis showed that the mRNA abundance of *AN4* was much higher in the corolla tube than in the anther or limb, whereas the mRNA abundance of *DPL* was much higher in the limb and corolla tube than in the anther (Fig. 2a). Notably, within the corolla tube, the transcript level of *AN4* was higher on the venation side than on the nonvenation side after the opening of the flower (Fig. 2b). The transcript level of *DPL* was higher on the venation side than on the nonvenation side and was higher than the expression of *AN4* on the nonvenation side (Fig. 2b).

Going further, we then investigated the *AN4* and *DPL* expression levels during the flower development. Based on corolla tube length (0.5, 1.5, 2.5, and 3.5 cm), flower development was divided into stage 1 (S1), stage 2 (S2), stage 3 (S3), and stage 4 (S4; Fig. 2c). During flower development, the venation pattern was gradually accentuated by the presence of anthocyanin. The mRNA abundance of *DPL* continuously decreased from S1 to S4 (Fig. 2d). However, the expression level of *AN4* continuously increased from S1 to S4 (Fig. 2d), in accordance with the emergence of venation. These results indicated that *AN4* could determine corolla tube venation in MD.

### *AN4* promotes anthocyanin biosynthesis in MD

**Fig. 3:**
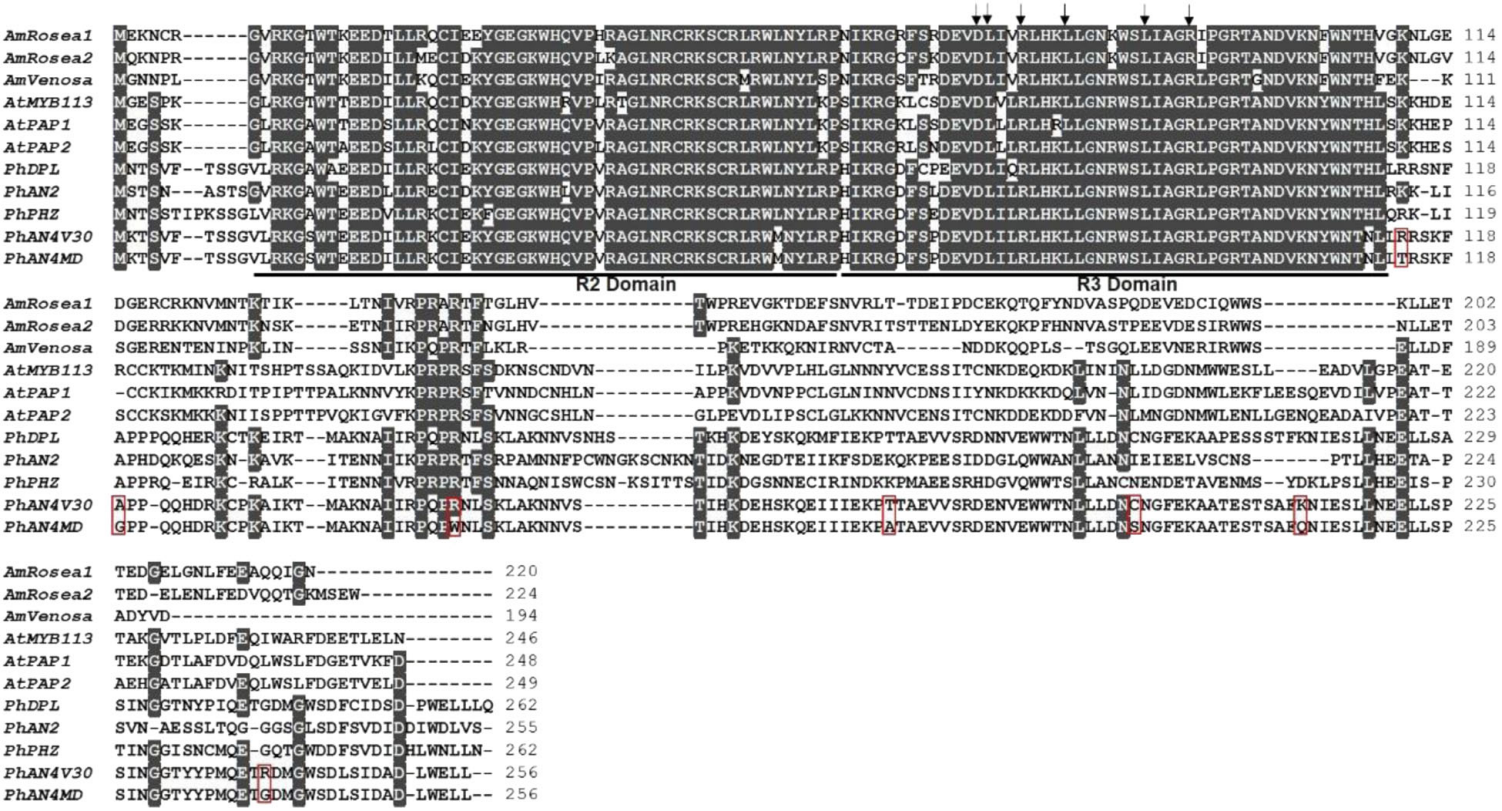
Alignment of the PhAN4^MD^ protein sequence with other anthocyanin-related R2R3-MYB transcription factors. The conserved R2 and R3 domains are indicated by solid lines. Vertical arrows indicate the bHLH-interacting motif ([D/E]Lx2[R/K]x3Lx6Lx3R) in the R3 domain. Red boxes indicate different residues between the AN4^V30^ and AN4^MD^ protein sequences

To confirm that *AN4*^*MD*^ encoded a functional R2R3-MYB transcription factor in MD, the transcript of *AN4* was isolated from this plant’s corolla tube. Sequence analysis revealed that the open reading frame of *AN4*^*MD*^ was highly similar to that of *AN4*^*V30*^, which has been identified as an activator of anthocyanin biosynthesis in the V30 line^27^. The deduced amino acid sequence of *AN4*^*MD*^, along with other anthocyanin-related R2R3-MYBs in petunia, contained the conserved [D/E]Lx_2_[R/K]x_3_Lx_6_Lx_3_R motif in its R3 domain (Fig. 3), as required for interacting with the AN1 (bHLH) protein^21^. Outside the conserved R2 and R3 domains, several substitutions of amino acids were present in *AN4*^*MD*^ compared with *AN4*^*V30*^ (Fig. 3).

**Fig. 4:**
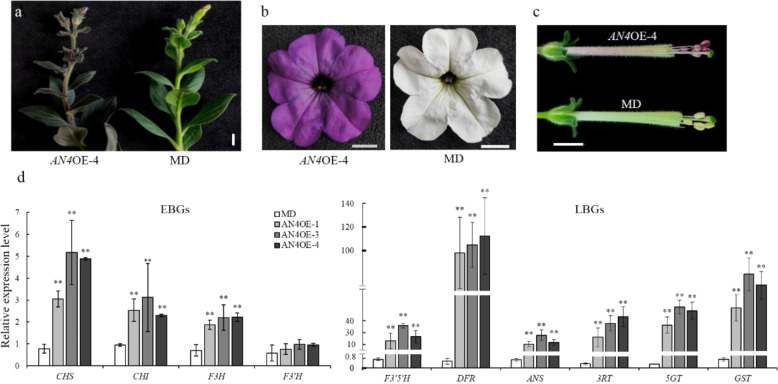
Functional analysis of *PhAN4*^*MD*^. **a** Branch of a representative line (*AN4*OE-4) compared with the MD line, scale bar = 1 cm. **b**, **c** Flowers from *AN4*OE-4 and MD, scale bars = 1 cm. **d** Transcript levels of early (*CHSA*, *CHSI*, *F3H*, and *F3*′*H*) and late (*F3*′*5*′*H*, *DFR*, *ANS*, *3RT*, *5GT*, and *GST*) anthocyanin biosynthetic genes in *AN4*OE lines and MD plants. *SAND* was used as the reference gene. Shown are the means ± SDs, *n* = 3. Asterisks indicate a significant difference (*P* < 0.01) from MD according to Student’s *t* test

To verify the function of *AN4*^*MD*^ in MD, transgenic lines expressing *AN4*^*MD*^ under the control of the CaMV35S promoter were generated in the MD background. Twelve independent transgenic lines (*AN4*OE) with pigmented leaves, stems, anthers, and corolla limbs were obtained (Fig. 4a, b). Although anthocyanins had accumulated in the anthers of these transgenic lines, their pollen was still yellow (Fig. 4c). The mRNA abundance of *AN4* in the five transgenic lines was assessed by qPCR. As expected, the transcription of *AN4* in these lines was higher than that in MD, and correspondingly, their anthocyanin content was markedly increased (Fig. S2a, b). We next investigated the effect of overexpressing *AN4*^*MD*^ on the mRNA abundance of anthocyanin biosynthetic genes. These qPCR results revealed that the expression levels of most of these genes, including *CHSA*, *CHSI*, *F3H*, *F3’H*, *F3’5’H*, *DFR*, *ANS*, *3RT*, *5GT*, and *GST* but not *F3*′*H*, were strongly upregulated in *AN4*OE transgenic plants compared with those in wild-type plants (Fig. 4d). These results indicated that AN4^MD^ functions as a transcriptional activator of the anthocyanin biosynthetic pathway in MD.

### *AN4* determines corolla tube venation patterning

**Fig. 5:**
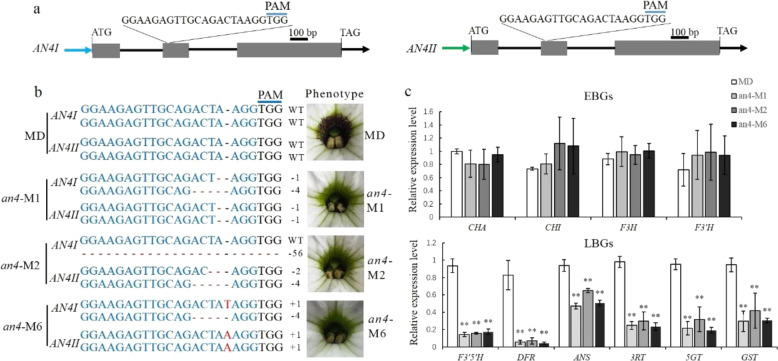
CRISPR/Cas9-mediated mutation in *AN4*. **a** Diagram of the target site in the genomic regions of *AN4I* and *AN4II*. The blue line indicates the PAM (NGG) motif. The gray boxes and black lines indicate exons and introns, respectively. **b** Targeted mutagenesis in *AN4I* and *AN4II* of the *an4*-M1, -M2, and -M6 lines and their corresponding phenotypes. The target sequence is shown in blue letters, for which red dashes indicate deletions, while red letters indicate insertions. **c** Transcript levels of early (*CHSA*, *CHSI*, *F3H*, and *F3*′*H*) and late (*F3*′*5*′*H*, *DFR*, *ANS*, *3RT*, *5GT*, and *GST*) anthocyanin biosynthetic genes in the *an4*-M1, -M2, and -M6 lines and MD. *SAND* was used as the reference gene. Shown are the means ± SDs, *n* = 3. Asterisks indicate a significant difference (*P* < 0.01) from MD according to Student’s *t* test

To identify the function of *AN4* in regulating corolla tube venation, the CRISPR/Cas9 system was applied to generate an *AN4* mutant. Referring to the genome sequence of *Petunia axillaris*^19^, two *AN4* genes, *AN4I* and *AN4II*, containing the same gene structure but different promoters were isolated from the MD genome (Fig. 5a). One sgRNA was designed to simultaneously target the second exon of both *AN4I* and *AN4II* (Fig. 5a); in this way, seven transgenic lines without venation patterning in their corolla tube were obtained. Three nonvenation lines (*an4*-M1, -M2, and *-*M6) were randomly selected to detect the mutation types in the *AN4I* and *AN4II* loci. In the *an4*-M1 and *an4*-M6 lines, biallelic mutations occurred in the *AN4I* and *AN4II* loci, and the *an4*-M2 line harbored a biallelic mutation of *AN4II* and a monoallelic mutation of *AN4I* (Fig. 5b). The indel mutations in these mutants resulted in frameshift mutations, rendering the *AN4* protein inactive. In the T1 lines of *an4*-M1, -M2, and -M6, venation patterning disappeared in the corolla tube as well. Considering that the target sequences of *AN4* and *DPL* were highly similar to each other, a potential off-target site was detected in the *DPL* locus of the *an4*-M1, -M2, and -M6 lines; however, no mutations could be found in the off-target site of these lines (Fig. S3b).

To investigate the role of AN4 in regulating the expression of EBGs and LBGs, qPCR was carried out to detect the relative expression levels of anthocyanin biosynthetic genes in the *an4*-M1, -M2, and -M6 lines. These results showed that transcript levels of EBGs (*CHSA*, *CHSI*, *F3H*, and *F3*′*H*) went unchanged, but those of LBGs (*F3*′*5*′*H*, *DFR*, *ANS*, *3RT*, *5GT*, and *GST*) were significantly downregulated in the *an4*-M1, -M2, and -M6 lines compared with those in MD (Fig. 5c). Corresponding to the nonvenation phenotype, the anthocyanin content was dramatically reduced in the *an4*-M1, -M2, and -M6 lines compared with that in MD (Fig. S2c). These results indicated that *AN4* preferentially governs LBGs to control the pigmentation of corolla tube venation.

## Discussion

Petunia is a classical model system for studying the biosynthesis and regulation of flower pigments^28–30^. Some pigmentation patterns in petunia flowers have been well characterized. For example, the spot pattern is the result of an unstable transposon insertion in the *ANTHOCYANIN3* locus^31^, and star-type and picotee patterns are induced by RNA silencing of *CHS*^32,33^. In this paper, we revealed that venation patterning in the corolla tube is determined by the R2R3-MYB transcription factor *AN4*, but not *DPL*.

Formerly, according to patterns of gene expression, segregation analysis, and vein-associated promoter activity, *DPL* was proposed to govern venation patterning in MD flower tubes^21^. However, the lines of evidence for this were indirect and inconclusive. First, the mRNA abundance of *DPL* gradually decreased during flower development (Fig. 2d), coinciding with the disappearance of vein-associated anthocyanin on the abaxial epidermis of the flower bud (Fig. S1h), whereas the venation patterning gradually emerged during flower development, coinciding with *AN4* expression (Fig. 2d). Second, despite the cosegregation of *DPL* and venation patterning, the latter’s formation may have been driven by *AN4* instead of *DPL* in the segregation analysis because *AN4* and *DPL* are on the same chromosome, lying just 60 kb apart^19^. Third, loss-of-function mutants or gene-silenced lines of *DPL*, which are important for verifying the specific roles of *DPL* in petunia, were lacking. Hence, overall, *DPL* might not determine venation patterning in petunia. By relying on the CRISPR/Cas9 system, we were able to obtain sought-after *dpl* mutants. In these, vein-associated anthocyanins on the abaxial epidermis of flower buds disappeared rather than venation patterning, which unchanged (Figs. 1d and S1). Combining the vein-associated promoter activity of *DPL*^21^ and the phenotype of CRISPR/Cas9-induced *DPL* mutants suggests that *DPL*’s role is to determine vein-associated anthocyanin patterning on the abaxial epidermis of flower buds. Nonetheless, vein-associated anthocyanin patterning was more pronounced under cold conditions (Fig. 1e) and matched well to *DPLpro*-directed GUS localization in the corolla^21^, thus suggesting that *DPL* may respond to cold stress in petunia.

To date, it has been reported that nine MYB genes—*AN4*, *AN2*, *DPL*, *PURPLE HAZE* (*PHZ*), *MYB27*, *MYBx*, *ANTHOCYANIN SYNTHESIS REGULATOR 1* (*ASR1*), *ASR2*, and *ASR3*—regulate anthocyanin biosynthesis in petunia^21,23,26,34,35^. These genes act as transcriptional activators or repressors of anthocyanin biosynthesis by positively or negatively regulating the expression of anthocyanin biosynthetic genes. Previous research has demonstrated that *AN4* determines the pigmentation of anthers in petunia, so *AN4* is primarily regarded as having recessive alleles in some petunia lines with acyanic anthers, such as those of MD, V26, R27, and W138 (refs. ^26,28^). However, we found that AN4 is a functional activator of anthocyanin biosynthesis genes in MD plants. The expression levels of most EBGs were upregulated in transgenic plants overexpressing *AN4* (Fig. 4d), but were not affected in CRISPR/Cas9-induced *an4* mutants (Fig. 5c). Similarly, the transcript levels of EBGs were not changed in the *an2* mutant^36^. In *Arabidopsis*, EBGs are activated by flavonol-specific R2R3-MYBs, including MYB11, MYB12, and MYB111 (refs. ^37,38^). The expression of EBGs in the *an2* mutant and CRISPR/Cas9-induced *an4* mutants might be activated by the orthologs of *AtMYB11*, *AtMYB12*, and *AtMYB111*, which have not been isolated in petunia. Therefore, it remains challenging to improve the regulatory mechanism controlling the expression of EBGs and LBGs in petunias.

During flower development, the spatiotemporal expression pattern of *AN4* was positively associated with the formation of venation patterning in the corolla tube (Fig. 2a, b, d). Furthermore, CRISPR/Cas9-induced *AN4* mutation resulted in the complete absence of venation patterning in MD corolla tubes (Fig. 5b). Together, these results indicate that *AN4* determines venation patterning in the corolla tube of MD flowers. In addition to regulating corolla tube venation, AN4 also regulates anther pigmentation^26^. Similar to *AN4*, *PeMYB12* determines not only venation patterning in sepals/petals, but also full pigmentation in the central lobe of the lip in *Phalaenopsis* spp.^15^. Chia et al. indicated that the dual functions of *PeMYB12* may arise from a differential regulatory mechanism that exists in different floral organs^15^. In the MD line, the expression of *AN4* was activated in its corolla tube but inactivated in its anthers (Fig. 2a), which indicates that *AN4* may be regulated via different mechanisms in the anther versus corolla tubes. The same expression pattern of *AN4* was also observed in the V26 line. The inactivation of *AN4* in the anthers of V26 was believed to result from the methylation that occurred in the *AN4* coding sequence and promoter, but the mechanism activating the expression of *AN4* in the corolla tube remains unclear^27^. Additional efforts are therefore required to dissect the upstream regulatory mechanism of *AN4*, which should be helpful for a better understanding of how *AN4* activity is governed to form venation patterning.

In addition to *AN4*, *Ve1* also participates in the regulation of corolla tube venation patterning in petunia. Unlike the phenotype of the *an4* mutants, a few longitudinal pigmented stripes appeared in the corolla tube of *ve1*^*−*^ individuals^20^, pointing to other as-of-yet unclear factors involved in determining corolla tube venation. Given the associations between *AN4* and *Ve* loci uncovered in genetic analysis^20,25^, elucidating the molecular nature of these *Ve* loci would provide insight into the mechanism underlying various venation patterns in petunia, making it a promising area of future research.

CRISPR/Cas9 is a powerful tool for creating null mutants and dissecting the functions of genes. To date, it has been used successfully to re-evaluate and redefine plant gene functions in a few cases^39–41^, from which novel insights were obtained. In the present work, the functioning of *DPL* and *AN4* was re-evaluated in petunia using the CRISPR/Cas9 system. The obtained results strongly suggest that *DPL* regulates vein-associated anthocyanin patterning on the abaxial epidermis of flower buds, while *AN4* determines corolla tube venation in petunia. These findings refine the regulatory framework of flower anthocyanin patterns in petunia and can facilitate further understanding of the mechanism underpinning these various patterns in petunias and perhaps other flowers.

## Materials and methods

### Plant materials and growth conditions

The petunia (*P. hybrida)* inbred species MD was used in this study. Transgenic and MD plants were grown under greenhouse conditions (16 h light/8 h dark photoperiod, 200–250 μmol m^−2^ s^−1^ light intensity, at 25 °C). For the cold treatment, the MD line and the *dpl*-M3, -M4, and -M5 mutants were cultured under greenhouse conditions until flowering, and the plants were placed in a growth chamber at 12 ± 2 °C for 2 weeks with a 16 h light/8 h dark photoperiod and 120 μmol m^−2^ s^−1^ light intensity.

### Vector construction and transformation of petunia

To isolate the *AN4* cDNA from MD, the gene-specific primers AN4^MD^-F/R were designed according to the sequence of *AN4*^*V30*^ (accession number HQ428105). Total RNA extraction and cDNA synthesis were performed, as previously described^42^. The complete coding sequence of *AN4*^*MD*^ was isolated from the cDNA of MD petals. The PCR products were cloned into a pMD19-T vector (Takara, Dalian, China) and then sequenced by Tsingke Biological Technology (Chongqing, China). To overexpress *AN4* in MD, the *p35S:AN4* vector was generated by cloning the coding sequence of *AN4* into a modified pGreenII0229 vector between the 35S promoter and 35S terminator. To generate *AN4*-knockout mutants, the genomic sequences of *AN4I* and *AN4II* were amplified from MD according to the genomic sequence of *P. axillaris*, and then, sgRNA targeting the border region of *AN4* exons was designed using the CRISPR-GE web tool (skl.scau.edu.cn)^43^. The sgRNA cassette was synthesized and cloned into a CRISPR/Cas9 construct to generate the pGGEAN4 vector, as described by Zhang et al.^44^. Likewise, to generate *DPL*-knockout mutants, a corresponding pGGEDPL vector was constructed as above. All the primers used for gene amplification or the construction of gene-editing vectors can be found in Supplementary Table 1. These recombinant vectors were introduced into *Agrobacterium tumefaciens* strain GV3101 by electroporation. The *Agrobacterium*-mediated transformation of petunia was performed according to Zhang et al.^44^.

### Detection of mutations

Genomic DNA was first extracted from transgenic lines via the CTAB method and then used as a template to amplify DNA fragments harboring the designed target sites with gene-specific primers (Supplementary Table 1). PCR was conducted using *TopTaq* DNA polymerase (Trans, Beijing, China) in a 25 μl volume. The amplification reaction was performed as follows: 94 °C for 5 min, followed by 28–30 cycles of 94 °C for 30 s, 55 °C for 30 s, and 72 °C for 2 min, and then 72 °C for 10 min. The PCR products were separated by 1% agarose gel electrophoresis and directly sequenced by Sanger sequencing. Then, the sequencing chromatograms were decoded into allelic sequences to identify the mutations using the CRISPR-GE web tool (skl.scau.edu.cn)^45^. The sequencing chromatograms are presented in Fig. S3.

### Real-time PCR analysis

Total RNA extraction and cDNA synthesis were carried out as previously described^42^. Quantitative real-time PCR was performed with Ssofast EvaGreen Supermix (Bio-Rad, Hercules, CA, USA) on a CFX96 Real-time PCR System (Bio-Rad, Hercules, CA, USA). Primers for anthocyanin biosynthetic genes were designed according to Albert et al.^34^. The *SAND* gene was used to normalize the samples^46^. Relative gene expression values were calculated using the methodology of Schmittgen and Livak^47^. The qPCR data are presented here as the mean ± SE of three biological replicates. All primer sequences used for qPCR are listed in Supplementary Table 1.

### Anthocyanin measurement

Extraction of anthocyanins was performed, as described by Viola et al.^48^. Briefly, 0.15 g fresh samples were ground into powder using liquid nitrogen and then incubated in 15 mL of 1% HCl–methanol (v/v) at 4 °C overnight in the dark. After centrifuging each sample, the absorbance of the resulting supernatant was measured at 530 and 657 nm on a Varioskan Flash Spetral Scanning Multimode Reader (Thermo Fisher, Waltham, MA, USA). The relative anthocyanin content was then determined as (*A*_530_ – 0.25 × *A*_657_) per gram fresh weight.

### Statistical analysis

The significance of differences among the data were determined by Student’s *t* test using SPSS 17.0 software. Statistically significant differences are indicated by asterisks * (*P* < 0.05) or ** (*P* < 0.01).

### Accession numbers

The sequences of the genes described in this work can be found in GenBank: *DPL*, HQ116169; *AN4*, HQ428104. The genome sequences of *AN4I* and *AN4II* can be obtained from the Sol Genomic Network (https://solgenomics.net/) under the accession numbers Peaxi162Scf00578g00008.1 and Peaxi162Scf00578g00007.1, respectively.

## Supplementary information

revised supplementary information
