# Supplementary material for: CRISPR/Cas9-mediated targeted mutation reveals a role for AN4 rather than DPL in regulating venation formation in the corolla tube of Petunia hybrida
Source: Hortic Res. 2021 Jun 1;8:116. doi: 10.1038/s41438-021-00555-6 (PMC8166957; doi:10.1038/s41438-021-00555-6)
Supplement: Supplementary file 1 — revised supplementary information [file 41438_2021_555_MOESM1_ESM.docx]

**Supplementary Figure S1**


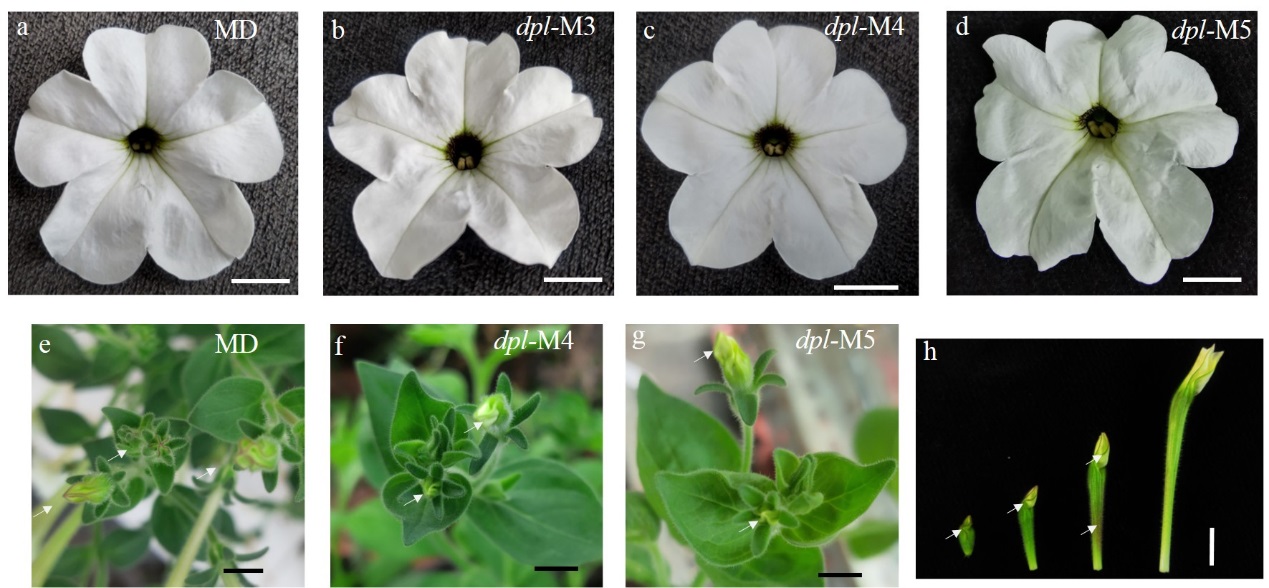


**Fig. S1** Floral anthocyanin patterns in MD and *dpl* mutants

**a-d:** Corolla tube venation in flower of *dpl* mutants and MD

**e-g:** Flower buds from MD, *dpl*-M4 and *dpl*-M5 mutants

**h:** Vein-associated anthocyanin pattern on abaxial surface of the flower bud of MD, arrows indicated the anthocyanin pattern

Scale bars = 1 cm.

**Supplementary Figure S2**


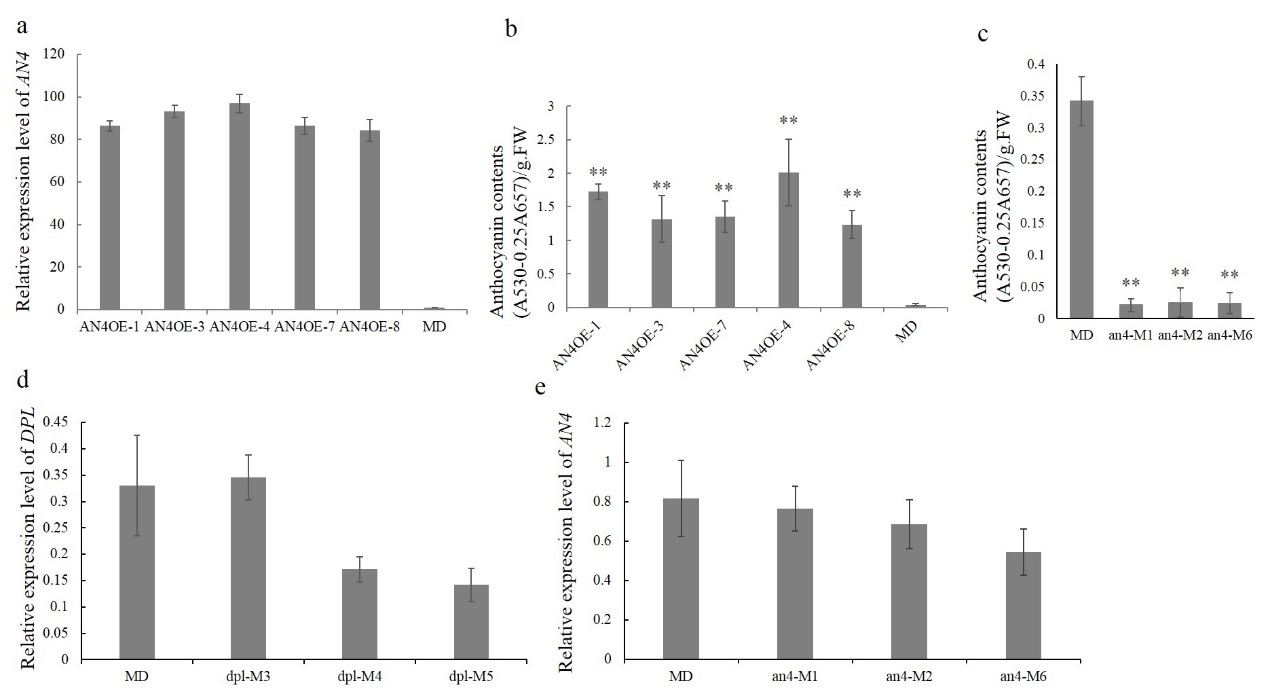


**Fig. S2** Anthocyanin content and expression level of *AN4* and *DPL* in transgenic lines and MD

**a:** Relative expression level of *AN4* in corolla tube of transgenic lines overexpressing *AN4*

**b:** Anthocyanin content in petal of transgenic lines overexpressing *AN4*

**c:** Anthocyanin content in corolla tube of *of an4*-M1*,* -M2 and –M6 and MD

**d:** Relative expression level of *DPL* in flower of *dpl*-M3, -M4 and –M5 and MD

**e:** Relative expression level of *AN4* in flower of an4-M1, -M2 and –M6 and MD

**Supplementary Figure S3**


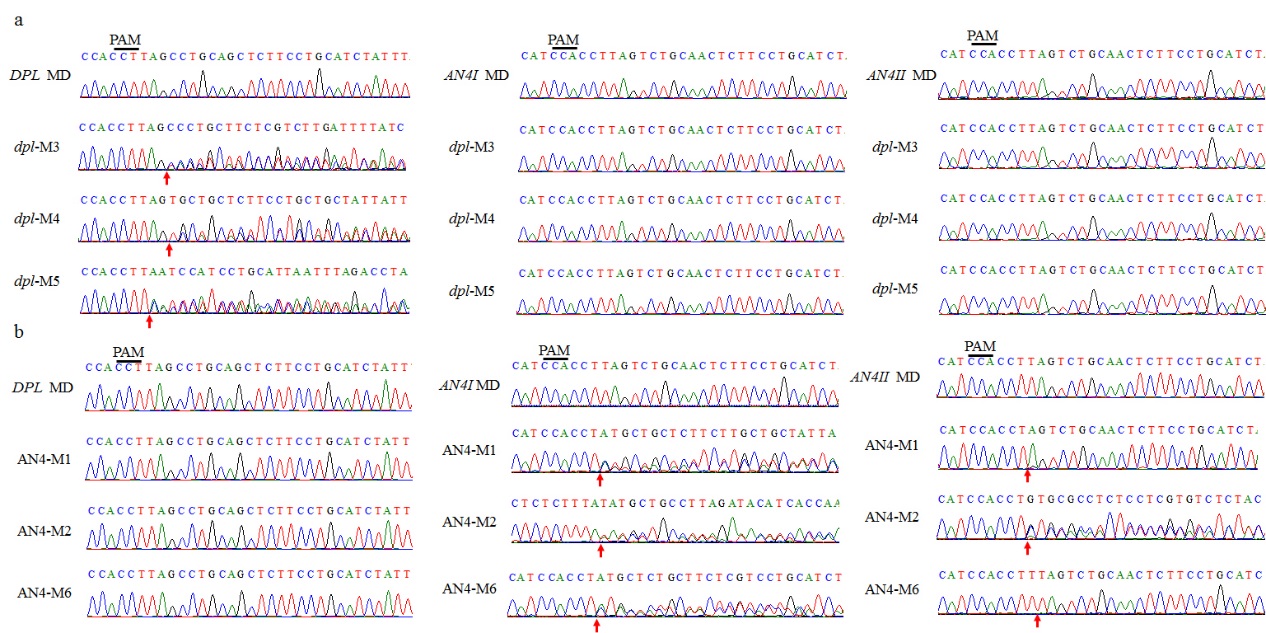


**Fig. S3** Sanger sequencing chromatograms of *DPL*, *AN4I* and *AN4II* fragments from *dpl* mutants (a) and *an4* mutants (b), respectively. Red arrow indicated the positions of mutation sites.

| Primer name | Sequence (5’→3’) | Comments | Length of  PCR product |
| --- | --- | --- | --- |
| AN4^MD^-F | cggaattcATGAAAACTTCTGTTTTTACGTCGTCG | For *AN4*-OE construction | 874 bp |
| AN4^MD^-R | cgggatccTAGACGCAGTTGTGCTTGG | For *AN4*-OE construction |  |
| DPL-sgF1 | GCAGGAAGAGCTGCAGGCTAgttttagagctagaaatagc | For pGGEDPL construction | 658 bp |
| DPL-sgR1 | TAGCCTGCAGCTCTTCCTGCaatcactacttcgactctag | For pGGEDPL construction |  |
| AN4-sgF1 | GGAAGAGTTGCAGACTAAGGgttttagagctagaaatagc | For pGGEAN4 construction | 658 bp |
| AN4-sgR1 | CCTTAGTCTGCAACTCTTCCaatcactacttcgactctag | For pGGEAN4 construction |  |
| DPL-F3 | GTGGCATCAAGTTCCTGTTAGAG | For *PhDPL* genomic DNA | 1377 bp |
| DPL-R3 | ACTAAAGTGGAGTGGGTTTCAAC | For *PhDPL* genomic DNA |  |
| AN4-1-F1 | CCAATGCTAATGGAGCTTAAGT | For *PhAN4I* genomic DNA | 1700 bp |
| AN4-1-R2 | TCCTAGAGGTCAGCATCAATAGA | For *PhAN4I* genomic DNA |  |
| AN4-2-F5 | CGCCTCCACAGTGTTGTCCAGTA | For *PhAN4II* genomic DNA | 2132 bp |
| AN4-R5 | TGAGAGGTTCCAAGGTTGAGGTCTT | For *PhAN4II* genomic DNA |  |
| DFR-A-QF | CCCCTAGTTTAATCACTGCCC | qRT-PCR | 290 bp |
| DFR-A-QR | GACCATCTTAGCCACATCGTAG | qRT-PCR |  |
| ANS-QF | TCTTCCATTGTGCTTTCCCTG | qRT-PCR | 80 bp |
| ANS-QR | GTTGCTGGAGTGTAGTCAGTAG | qRT-PCR |  |
| CHS-A-QF | ACATGGCACCTTCTCTTGATG | qRT-PCR | 119 bp |
| CHS-A-QR | GGTAATTTTGGACTTGGGCTG | qRT-PCR |  |
| GST-QF | GGTTGTGAAAGTGCATGGTTC | qRT-PCR | 92 bp |
| GST-QR | GGATAAGCTCAAAATCAACCCC | qRT-PCR |  |
| F3′5′H-QF | ATAGGCGTTTACTCGAATCCG | qRT-PCR | 89 bp |
| F3′5′H-QR | TGGTGTAGAAGGGTGTTTTCG | qRT-PCR |  |
| 3RT-QF | TGGAAAGAGTGAAAGACAAGGG | qRT-PCR | 227 bp |
| 3RT-QR | CCTATTAATCTCCACCCCAGC | qRT-PCR |  |
| F3'H-QF | AGCTGGACGTAGGATTTGTG | qRT-PCR | 167 bp |
| F3'H-QR | ATGGATCAGCCCGTTGTAAG | qRT-PCR |  |
| CHI-A-QF | TATGATCTTGCCCTTGACGG | qRT-PCR | 82 bp |
| CHI-A-QR | CGTTCCTATCCCCTTCCAATG | qRT-PCR |  |
| F3H-QF | CACCAGAGGCGATAGTGTATC | qRT-PCR | 214 bp |
| F3H-QR | GCAAGAATTTCCTCAATGGGC | qRT-PCR |  |
| 5GT-QF | TTGCCGTGATATTACTTCCCC | qRT-PCR | 83 bp |
| 5GT-QR | TGTCTTAGCACATTCCCTCAG | qRT-PCR |  |
| AN4-QF | GGATAACAGCAACGGATTTGAGAAGG | qRT-PCR | 174 bp |
| AN4-QR | AGGTCAGCATCAATAGAAAGGTCACT | qRT-PCR |  |
| SAND-QF | CTTACGACGAGTTCAGATGCC | qRT-PCR | 135 bp |
| SAND-QR | TAAGTCCTCAACACGCATGC | qRT-PCR |  |

**Supplementary Table S1** Primers used in this study
